# Attitudes of midwifery students towards teaching breast-self examination

**DOI:** 10.2478/v10019-010-0009-9

**Published:** 2010-03-18

**Authors:** Andrej Plesnicar, Martina Golicnik, Irena Kirar Fazarinc, Bozo Kralj, Viljem Kovac, Blanka Kores Plesnicar

**Affiliations:** 1 Faculty of Health Sciences, University of Ljubljana, Ljubljana, Slovenia; 2 Student Health Center, Ljubljana, Slovenia; 3 School of Health Science, Novo Mesto, Slovenia; 4 Department of Radiotherapy, Institute of Oncology, Ljubljana, Slovenia; 5 Faculty of Medicine, University of Ljubljana, Ljubljana, Slovenia

**Keywords:** midwifery students, breast cancer, teaching breast self-examination, breast health awareness, specific education

## Abstract

**Background:**

The purpose of this study was to asses the attitude of undergraduate midwifery students towards teaching other women in methods of breast self-examination (BSE).

**Participants and methods.:**

The study was performed at the beginning and at the end of students’ study at the Faculty of Health Sciences in Ljubljana, Slovenia. It was carried out during the academic year 2002/2003 and involved 28 first and 25 third year undergraduate midwifery students. The data were gathered from questionnaires and processed with the use of descriptive and inferential statistics.

**Results:**

All study participants were of the opinion that teaching other women in methods of BSE is of great importance for an early detection of breast cancer (BC) and that this task ought to be one of their duties. There were no significant differences between the two groups when the readiness to upgrade their own knowledge of BSE or when the optimism regarding the progress in breast cancer detection and therapy in the future were concerned.

**Conclusions:**

The readiness of midwifery students to pass the knowledge of BSE to other women could help to increase their breast health awareness and thus improve their willingness and ability to detect early changes, associated with BC.

## Introduction

Breast cancer is the most common form of cancer and the cause of death from cancer in women in Slovenia (population two million), where it was diagnosed in 1112 women in the year 2006 and has shown a fivefold increase in its incidence in the last five decades.^1^–^3^ Breast cancer incidence may be low in less developed countries, but survival rates of those suffering from this disease are also low, thus making it an important health care problem all over the world.^4^,^5^

The efficacy of BC screening with mammography has been confirmed in a number of randomized and observation studies.^6^,^7^ However, small palpable tumours can also be detected with the clinical breast examination (CBE), performed in clinical surroundings, and with BSE, which can be done by properly trained women themselves.^6^–^8^ Despite many controversies, it has been accepted that BSE increases the awareness of normal appearance and structure of breasts and the consequent ability to detect subtle changes that don’t correspond to the normal features of healthy breasts. Breast health awareness may, therefore, increase the ability of women to find small palpable tumours early, leading them to consult their physician immediately after discovering undue changes in their breasts.^6^,^9^,^10^ Unfortunately, few women perform BSE and there is little information on the attitude of individual women and specific groups of women towards this way of breast change detection.^3^,^6^

In this study, we, thus, assessed the attitude of midwifery students towards the teaching other women in methods of BSE. We also assessed how the specific education of midwifery students, gained during the course of their three year study, affects their attitude towards BSE. Finally, we examined their attitude towards the progress in BC detection and treatment in the future.

## Participants and methods

Twenty eight first year and 25 third year midwifery students were included in the study carried out in the academic year 2002/2003 at the Faculty of Health Sciences in Ljubljana, Slovenia. There were no males among the midwifery students in both study groups in that academic year. The part of the study involving the first-year students was carried out at the end of their first term and the part involving third-year students at the end of their sixth term. A written consent was obtained from all the involved participants before enrolling them in the study and all were asked to complete the questionnaires with data about their personal characteristics. Questionnaires also included five statements concerning their attitudes whether teaching other women in methods of BSE helps in BC detection, whether teaching other women about BSE ought to be one of their duties, about their attitude regarding additional BSE training and the form of such training. Finally, they were asked to express their attitude about the development and successfulness of the BC treatment in the future.

The acquired data were sorted and analyzed with the help of Microsoft Excel 97 and SPSS 11.0 for Windows programs. Most answers were assessed with the use of Chi-square test for 2X2 contingency tables with which the differences in frequencies were verified. The results were considered as statistically significant if p<0.05.^11^

## Results

The mean age of the first-year midwifery students in the study was 19.86 ± 1.18 (SD) years (range 18–23 years), the mean age of the third-year students was 21.76 ± 2.26 (SD) years (range 20–32 years). None of them had undergone a mammography or ultrasound breast examination prior to participating in the study.

All the participants, regardless of the year of study, were of the opinion that teaching BSE to other women helps in the early detection of BC. All were of the opinion that teaching and informing women of the advantages and disadvantages of BSE ought to be one of their duties. Most of the participants from both groups would be willing to receive additional training in how to perform BSE. The number of the participants willing to receive this training was slightly smaller in the group of the third-year students, but the difference was not statistically significant. In the group of the first-year students more participants were in favour of practical BSE lessons given live by experts and demonstrators as compared to a brochure on BSE obtained by mail (Table 1). The majority of the participants from both groups were optimistic in their opinion regarding the progress in the development and successfulness of the BC detection and the treatment in the future. The number of participants holding such opinion was slightly larger in the group of the third-year students, the difference between both groups was not statistically significant (Table 1).

## Discussion

In this study, we found that midwifery students could be regarded as having a positive attitude towards teaching other women in methods of BSE. They were of opinion that this type of teaching helps in the BC detection and that it ought to be one of their duties. Most of the participants were also willing to receive the additional training in BSE themselves. The opinion that they should play an active part in educating other women and that they should additionally educate themselves probably makes them optimistic in their views of the development and successfulness of the BC detection and the treatment in the future.

A favourable attitude of midwifery students towards BSE may come from an inherent motivation to become involved in problems associated with BC and from the specific education they gain during the course of their study. This knowledge makes them more informed than other women^5^,^12^–^18^, giving them a profound insight into the anatomy, histology and physiology of healthy breasts, as well as into the significance of the incidence, mortality and pathogenic characteristics of BC.^19^ Midwifery students are at the end of their study in possession of an important amount of information about BC and know that it is an important public health problem. In this context, it is safe to conclude that their specific education is of utmost importance.

A special emphasis should be given to the attitude of all the participants in the study that BSE is important in the early detection of BC. All of them would fully participate in teaching other women about BSE that certainly must include its advantages and disadvantages.^6^,^15^ Their willingness to teach BSE should receive the additional support by giving the results of the studies investigating BSE some further consideration. According to some of the studies, BSE does not decrease the mortality caused by BC.^20^–^22^ However, the results of one of such studies also show that the compliance of one of the study groups to BSE program (BSE every month or every two months) helped to achieve a higher rate of 15 year survival.^22^ Some of the studies also show that BSE reduces the size of newly discovered primary breast tumours^20^,^23^,^24^, which may lead to a prompter use of modern diagnostic procedures and enables carrying out more conservative surgical procedures.^10^,^25^ Even when examining the negative results from some of these studies, a careful consideration should be given to local cultural values, level of readiness of participants to perform BSE and the likelihood that all of them were properly informed about BC issues.^20^,^26^–^29^

Despite some of the doubts mentioned above, the possibility that BSE is used for achieving the increased breast health awareness in women should be examined carefully.^6^,^30^–^32^ The breast health awareness means accepting health responsibilities to a greater extent by being able to recognize a normal appearance and structure of breasts during different cycle periods and with regard to age, by being able to recognize undue changes and to inform the physician immediately.^6^,^23^,^30^,^31^,^33^ If the breast health awareness in women is to increase, the easiest way to achieve this would probably be by teaching them how to perform BSE. In some opinions the breast health awareness should take the role of BSE^30^, with BSE itself being not much different from what the concept of the breast health awareness includes.^23^,^34^

The majority of midwifery students in our study were willing to receive the additional education in relation to BSE. Most of them favoured the practical demonstration of the BSE procedures. A smaller interest in the additional training and practical demonstrations is understandable with the students finishing their study and, on the other hand, this smaller interest again proves the importance of knowledge gained during the study of midwifery.^19^ A slightly bigger number of those who hold an optimistic view of the future of the BC treatment in the group of the third-year students probably also points to the importance of this knowledge.^18^,^35^,^36^

In this study we found that female midwifery students included in the study have a favourable attitude towards teaching BSE, although it is undoubtedly hard to learn and teach.^37^–^39^ All considered BSE to be of great importance for an early breast cancer detection and all were willing to teach it to other women, including those with previously treated malignant disease.^40^ As a consequence of their attitude towards BSE and specific contents of their study, midwifery students could towards the end of their study probably also perform CBE quite efficiently and relieve other women of difficult endeavour of learning all the steps of the appropriate and effective BSE.^41^,^42^ In Slovenia and elsewhere, this application of their knowledge would be especially important when younger women are in question, because in their cases mammography screening is not so efficient.^9^,^23^ On the other hand, the participants in this study could, due to their knowledge and other characteristics, represent a specific group continuing with quality and regular BSE. Their attitude towards and knowledge of BSE could, in due course, be evaluated again. However, in some of the less developed parts of the world, the knowledge of BSE could enable similarly educated midwives to have an important impact on the early detection of breast cancer and, along with other nurses, an important role in health promotion, prevention and education activities associated with breast cancer.^43^,^44^

In conclusion, in this study we show that midwifery students, a highly motivated and specifically educated group, are ready to pass the knowledge of BSE to other women, to receive the further BSE education themselves and are generally optimistic about the BC detection and the treatment in the future. In this way, they could have an important role in increasing the breast health awareness of other women and thus improve their willingness and ability to detect early changes associated with BC.

## Figures and Tables

**TABLE 1:** The attitudes of midwifery students (Faculty of Health Sciences, Ljubljana, Slovenia, academic year 2002/2003) towards teaching BSE, receiving additional BSE education and breast towards BC detection and treatment in the future

		**1. year (n = 28)**	**3. year (n = 25)**	**Chi-square**	**p-value**
Teaching BSE helps early BC detection	Yes	28 (100%)	25 (100%)	/	/
	No	0 (0%)	0 (0%)		
Teaching BSE should be one of midwives’ duties	Yes	28 (100%)	25 (100%)	/	/
	No	0 (0%)	0 (0%)		
I am willing to receive further BSE education	Yes	26 (92.86%)	22 (88%)	0.362	0.5469
	No	2 (7.14%)	3 (12%)		
I favour practical demonstration of BSE	Yes	22 (78.56%)	16 (64%)	0.9327	0.3341
	No	6 (21.44%)	9 (36%)		
I am optimistic about BC detection and treatment in the future	Yes	24 (85.71%)	23 (92%)	0.5311	0.4664
	No	4 (14.29%)	2 (8%)		

n = number; BSE = breast self-examination; BC = breast cancer
